# Follow-up of telemedicine mental health interventions amid COVID-19 pandemic

**DOI:** 10.1038/s41598-024-65382-w

**Published:** 2024-06-28

**Authors:** Carlos Roncero, Sara Díaz-Trejo, Esther Álvarez-Lamas, LLanyra García-Ullán, Miriam Bersabé-Pérez, José Antonio Benito-Sánchez, Armando González-Sánchez

**Affiliations:** 1 Psychiatric Unit, School of Medicine, University of Salamanca (Spain), Campus Miguel de Unamuno, Calle Alfonso X El Sabio s/n, 37007 Salamanca, Spain; 2 Instituto de Investigación Biomédica de Salamanca (IBSAL), Hospital Virgen de la Vega, 10ª Planta, Paseo de San Vicente, 58-182, 37007 Salamanca, Spain; 3 Psychiatry Service, University of Salamanca Health Care Complex, Paseo de San Vicente 58-182, 37007 Salamanca, Spain; 4 Psiquiatric Service, Hospital Infantil Universitario Niño Jesús, Av. de Menéndez Pelayo, 65, Retiro, 28009 Madrid, Spain; 5 Facultad de Psicología, Universidad Pontificia de Salamanca (UPSA), C/ Compañía, 5, 37002 Salamanca, Spain

**Keywords:** COVID-19, Healthcare, Mental health, Spain, Telemedicine, Human behaviour, Public health

## Abstract

The initiation of the program Mental Health Support Program for Coronavirus Infection addressed the increased demand for mental health services in the province of Salamanca, resulting from the COVID-19 pandemic. The psychiatry service provided care for COVID-19 patients, their families, and healthcare workers who treated them, as these groups were identified as being at risk. This study aims to describe the assistance provided, including personnel and resources utilized, types of interventions carried out, and to assess the demand for mental health care and predominant symptoms and emotions experienced by patients. Billboards and the complex’s intranet publicized the program. Specific clinical approach using telemedicine were provide from March 2020 to December 2021 to COVID-19 patients, their relatives, and healthcare workers. 216 patients were included with a mean age of 53.2 years, with women comprising 77.3% of this group. All the groups received treatment in similar proportions. Over a period of 730 h, a total of 1376 interventions were performed, with an average duration of 31.8 min per intervention. The program could treat 79.6% of these patients without requiring referrals to other services. When the program concluded, only 21 participants (9.7%) were discharged to the local mental health network to continue their mental health treatment. The program effectively reduced the burden on regular mental health services due to its ability to treat most patients without requiring referrals. The program was able to attend to most mental health requests with minimal involvement of the regular mental health service.

## Introduction

In December 2019, Spain issued a COVID-19 alert. During this time, transportation was restricted, a lockdown was implemented, and only essential economic activities were permitted^1^. In 2020, COVID-19 accounted for 28.3% of total deaths, with 60,358 registered deaths. In Castilla y León region, the Odds Ratio (OR) of dying from COVID-19 compared to any other cause was 2.97 (95% CI 2.87–3.08)^2^.

Prolonged exposure to traumatic events and stressors can significantly impact psychological health. Previous studies on COVID-19 epidemic alert have shown that these events can affect the mental health of populations and may result in conditions such as Post-Traumatic Stress Disorder (PTSD), anxiety, or depression^3,4^. In Spain, cases of depression, anxiety, and PTSD increased during COVID-19 alert^5^. These conditions disproportionately affected women who had family members infected with COVID-19 or who had a previous diagnosis of mental illness^6^. Additionally, there was widespread fear of contracting the disease and uncertainty about the future as the number of infections and deaths continued to rise.

Among patients with COVID-19, evidence shows a prevalence of 45% for depression (95% CI 37–54%), 47% for anxiety (95% CI 37–57%), and 34% for insomnia (95% CI 19–50%)^7^. Healthcare workers^8^ and patients themselves^9^ are among the most vulnerable groups. Compared to pre-pandemic levels, healthcare workers have reported increased anxiety (OR 3.03; 95% CI 2.53–3.62), increased substance use (OR 5.74; 95% CI 2.53–13.03), and greater burnout from patient care (OR 5.19; 95% CI 3.61–7.46)^8^. Insomnia, anxiety, and depression have been common symptoms among healthcare workers^10^, with some individuals even experiencing suicidal thoughts^11^ rising suicide rates and increased utilization of mental health services^12^. Furthermore, a relationship between mental health and increased mortality among COVID-19 patients has been established^9^.

The pandemic has also impacted the relatives of individuals affected by COVID-19^13^. Living with someone infected with COVID-19 has been identified as a risk factor for mental health problems, indicating that the pandemic has had consequences for both the physical and mental health of family members or those who care for them^11,12^. The pandemic has necessitated the reorganization of healthcare services, with consequences for the well-being of healthcare professionals^10,14^. Alongside the COVID-19 pandemic, there has also been a surge in psychiatric illness^15^.

It has been claimed that early psychological intervention and treatment can help mitigate the effects of PTSD, anxiety, depression, and other chronic psychological conditions among various population groups^11^. Due to limitations on in-person contact resulting from the risk of contagion, telemedicine has been successfully implemented and has proven effective in addressing mental health needs during the COVID-19 pandemic^16^. As a result, specific mental health programs targeting vulnerable populations are needed, trying to avoid overbooking of the regular mental health service^17,18^.

To address the increased demand for mental health services, Salamanca initiated the Mental Health Support Program for Coronavirus Infection called PASMICOR. This study aims to evaluate the PASMICOR program as a whole, from its inception to its conclusion. A previous manuscript was published with data from the first 9 months because we deemed it highly important to inform other hospitals about the specific program being implemented, so they could replicate it. However, this current article encompasses the program in its entirety: from inception to conclusion, emphasizing the effectiveness of the complete program. The general objectives of this study are to describe the mental health care response provided to COVID-19 patients, their family members, and healthcare professionals over the 18-month duration of the program; to assess whether the program was able to meet demand during a national emergency without requiring referrals; to evaluate the number of psychology professionals and proportion of users per population in the province of Salamanca; and to assess patient outcomes and overall program effectiveness. The specific objectives are to identify the most common symptoms and emotions experienced by patients; to determine the types of interventions used; to assess the personnel and time required to meet demand; and to evaluate the effectiveness of telepsychology as a practical form of assistance.

## Methods

The initiation of the PASMICOR program occurred during a peak in hospitalizations in March 2020 and concluded at the end of December 2021. The PASMICOR program was established with the primary purpose of preventing adverse mental health outcomes that could arise from the pandemic, confinement, and social alarm. By providing care for individuals who might otherwise overwhelm mental health services, the program was strategically designed to alleviate the strain on mental health network.

To be eligible for the PASMICOR program, individuals had to meet certain criteria. They had to be either a COVID-19 patient, a family member of a COVID-19 patient, or a healthcare professional caring for COVID-19 patients. Additionally, they could not be currently receiving treatment for a mental disorder from another healthcare professional, both to avoid interfering with their ongoing treatment and to align with the program's objectives of addressing the needs of individuals underserved regarding COVID-19-related issues. Furthermore, they could not have an acute illness, and had to have the ability to communicate with medical personnel via speaking Age was not a criteria selection. Eligible individuals also had to provide their express consent to participate in the program. Informed consent was obtained from all the study participants and/or their legal guardians. Individuals residing outside the province of Salamanca were excluded from participation.

Advertising posters were displayed in common areas of the University of Salamanca Health Care Complex and the University of Salamanca Healthcare Complex intranet disseminated information about the program. Primary Care Management and professional associations for nursing homes also promoted the program.

The professional attending to COVID-19 patients offered specialized mental health care services to both the COVID-19 patients and their families, provided they met the inclusion criteria. If they accepted the treatment, a request was generated via email. Health professionals could also access this service, provided they sent an internal email with the request themselves. These requests were received by a clinical psychologist who conducted a personalized evaluation process to classify, and triage based on factors such as symptom severity and type of user (e.g., COVID-19 patient, family member, or healthcare worker). This information was used to assign each patient to an appropriate mental health professional who could best address their needs. Once assigned, patients received telephone-based interventions from their assigned psychologist.

A standardized document was used to record each patient’s coded identification information while anonymizing their personal data (see supplementary material). This registry also included information on sociodemographic variables (e.g., sex, age, marital status, profession, employment status), group membership (e.g., COVID-19 patient, healthcare worker or family member), group specifications (e.g., hospitalization status or type of isolation), level of complexity (with two levels differentiated by symptom severity: Level I for less complex cases and Level II for cases requiring calls lasting more than 20 min or involving intense emotional expression or worrisome symptoms), predominant symptoms and emotions experienced by patients; type of intervention carried out; patient outcomes; number and duration of telephone interventions. All professionals who completed the template form were adequately instructed in specific training for its proper completion.

Psychologists specializing in clinical psychology conducted this single-center study during the COVID-19 pandemic. The program implemented a new approach to treatment by transitioning from face-to-face to remote consultations using telemedicine. During PASMICOR’s implementation, short-term, problem-focused telephone interventions were coordinated with the aim of producing rapid and effective change in patients. There were 12 part-time clinical psychologists and 2 part-time Resident Internal Psychologists, while 4 full-time psychiatrists were available to provide care for second-level users. At the sixth month, three more clinical psychologists were recruited to strengthen the program and the part–time psychologist came back to their usual works and clinics.

After 9 months of data collection, an interim study was analysed and showed that fear was a predominant emotion among healthcare workers while sadness and feelings of helplessness were more common among patients. Fear and sadness were also prevalent among family members^17^. The most effective treatments included techniques related to emotional expression and regulation as well as self-care. Mindfulness was particularly effective among healthcare professionals while grief counselling and providing information about available resources were beneficial for family members^17^. After 18 months of follow-up the program was closed, and no new patient were admitted since October. Patient who needed more follow-ups were referred progressively to the Salamanca mental health network until December 2021 this study analysed all available data and evaluated the overall effectiveness of the PASMICOR program.

### Data analysis

To assess the impact of the program on hospital burden detailed information about these tests, along with the data collection questionnaire, is included in an appendix document (see supplementary material) for reference. This analysis employs data gathered through the questionnaire detailed in the supplementary material, which provides a comprehensive overview of the variables considered and the statistical tests applied. The analysis and reporting of sociodemographic variables of program participants will be as frequencies and percentages. Median, mean, and standard deviation statistics will describe intervention characteristics. A frequency table showing the characteristics of program participants will present patient outcomes.

Analyses were conducted on the data collected in the supplementary material. For the analyses, the number and duration of calls, levels, and other variables recorded were related. Please refer to the attached document.

Pearson correlations will be calculated and reported along with their corresponding p-values. To analyze percentage increases, contingency tables were constructed using Kramer’s χ^2^ and Phi and V statistics. The calculation of corrected residuals identified significant differences between cells; values outside ± 1.96 were considered significant. SPSS performed statistical analyses^19^. Non-parametric tests such as the Mann–Whitney U test and the Kruskal–Wallis test were employed in our analysis.

Finally, a profile of program participants will be presented to determine whether there are differences in call duration or frequency based on factors such as gender, age, or type of user. A *p*-value < 0.05 will be considered statistically significant for these analyses.

### Ethical standards

This project was approved by the Bioethics Committee of the University of Salamanca Health Care Complex under the code: GRS59/A/20. This study was conducted in accordance with the guidelines of the Declaration of Helsinki, the appropriate measures have been taken to guarantee the complete confidentiality of the personal data of those participating in this study and in accordance with the "Organic Law 15/1999 of 13 December on the Protection of Personal Data" of the Government of Spain, under the protection of Law 41/2002 and in particular article 6.2 on the use of clinical documentation.

## Results

### Participant principal characteristics

A total of 216 individuals were included in the program, with an average age of 53.92 years and ranged in age from 10 to over 90 years old, with the majority being between 51 and 60 years old. Of these participants, 167 (77.3%) were women. Most participants were either employed or unemployed at the time of enrolment. Similar proportions of healthcare workers and individuals affected by COVID-19 received care through the program, with slightly fewer family members receiving care. Table 1 presents the frequencies of participant characteristics.

**Table 1:** Frequencies of participant characteristics by marital status, profession, employment situation, and type of user.

Marital status	n	Profession	n	Employment situation	n	Type of user	n
Single	32	Doctor	14	Active	71	Health worker	78
Coupled	145	Nurse	33	Sick leave	65	Patient	76
Widowed	28	Clinical assistant	28	Retired	38	Relative	62
Separated	8	Other healthcare environment profession	17				
		Others	79				
Lost data	3	Lost data	45	Lost data	44	Lost data	0
Total	213	Total	195	Total	196	Total	216

### Type of user: health workers, patients and/or relatives

In terms of group membership, healthcare workers made up 36.1% (78 participants) of the program, COVID-19 patients made up 35.2% (76 participants), and relatives of COVID-19 patients made up 28.7% (62 participants). The proportion of healthcare workers, COVID-19 patients or family members participating in the program did not change significantly over time. All groups received similar proportions of interventions (*p* = 0.491). However, healthcare workers categorized as level-2 received 24.9% more interventions than family members (*p* = 0.004). Despite family members receiving twice as many interventions as COVID-19 patients and 1.4 times more than healthcare workers, there were no significant differences in the number (*p* = 0.071) or duration (*p* = 0.091) of calls between groups.

### Evolution

Of the patients who met their treatment objectives and were discharged, 5 were readmitted to the program for continued care: 3 during the first 9 months and 2 during the second 9 months. An additional 5 patients did not continue with the program due to death. The program ran for a duration of eighteen months, plus two additional months for discharge period; during which we provided assistance to all participants who sought it. The program ensured that all participants received complete care. 21 participants (9.7%) were referred to the Salamanca mental health care network.

### Temporal correlation: hospital burden and PASMICOR admissions

**Figure 1:**
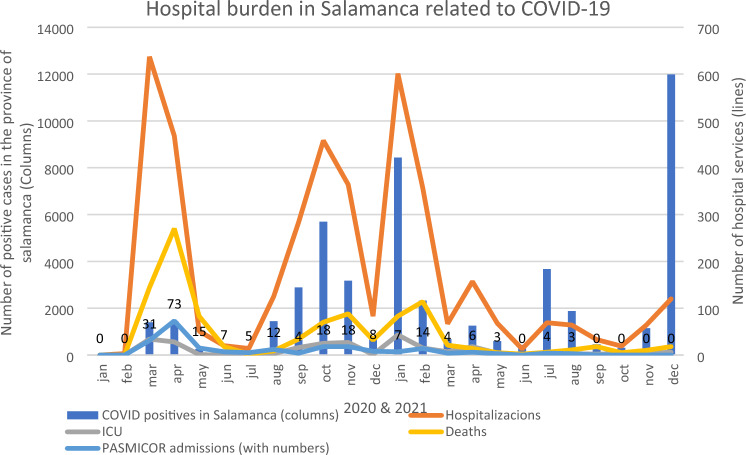
Comparison of COVID-19 Incidence and Hospital Care Services in the Province of Salamanca with Admissions to the PASMICOR Program. *ICU* Intensive Care Unit. Data obtained from^1^.

There was no significant correlation between PASMICOR admissions and the number of positive COVID-19 cases in Salamanca (r = 0.392, *p* = 0.058). However, PASMICOR admissions moderately correlated with admissions to the Intensive Care Unit (r = 0.533, *p* = 0.007) and strongly correlated with hospitalizations (r = 0.737, *p* < 0.001) and deaths (r = 0.775, *p* < 0.001) due to COVID-19 (Fig. 1).

### Intervention characteristics results

We conducted a total of 1376 interventions over the course of the PASMICOR program, totalling 730 h of telephone-based care. Each intervention lasted an average of 31.8 min. The duration and frequency of interventions varied considerably, ranging from brief, single interventions lasting 10 min to more extensive treatment involving up to 38 interventions and over 27.5 h of telepsychology assistance. From admission to discharge, each patient required a median of 4 calls (mean = 6.36, SD = 6.37) and a median of 120 min of telephone-based care (mean = 202.73, SD = 241.83).

Women received more interventions (mean = 7.06, SD = 6.88) and longer total intervention duration (mean = 234.97, SD = 265.23) than men (mean = 4.60, SD = 4.06; *p* = 0.016 and mean = 119.91, SD = 114.67; *p* < 0.001, respectively). Additionally, individual intervention sessions lasted longer for women, with an average duration of 32 min compared to 26 min for men (*p* = 0.005). Women also received 18.3% more level-2 interventions than men (*p* = 0.012).

The age distribution of program participants was not normal (*p* < 0.001). Most participants were between 51 and 60 years old (27.8%). On average, healthcare workers were younger than COVID-19 patients or their family members (*p* < 0.001). Age group was not significantly associated with the number of calls received by participants (*p* = 0.604) or with call duration (*p* = 0.166). Among participants aged 81–90, the number of level-1 interventions was lower than expected (Z_fixed_ = − 2.1). Notably, the majority of participants (79.6%) received comprehensive care through the program, 9.7% were referred to Psychiatry or Psychology services (where they receive mental health face to face treatment as suggested by the professionals in charge) and 6% refused treatment or deceased and a 4.6% of missing data. User characteristics can be seen in Table 2.

**Table 2:** Patient outcomes by participant characteristics.

User characteristics							Lost data	Total
		Discharge	Refused	Exitus	Referral			
					Psychiatry	Psychology		
Sex	Male	42	2	3	2	0	–	49
	Female	140	6	2	6	13	–	167
	Total	172	8	5	8	13	10	216
Age	0–10	1	0	0	0	0	–	1
	11–20	0	0	0	0	0	–	0
	21–30	11	1	0	0	1	–	13
	31–40	24	0	0	2	3	–	29
	41–50	33	2	0	2	4	–	41
	51–60	52	1	0	2	3	–	58
	61–70	28	3	2	1	1	–	35
	71–80	20	0	1	1	1	–	23
	81–90	5	0	2	0	0	–	7
	> 90	2	0	0	0	0	–	2
	Total	167	7	5	8	13	16	200
Origin	USHC	23	1	0	2	6	–	32
	Primary care	7	1	0	0	0	–	8
	Elderly home	2	0	0	0	0	–	2
	Prison	1	0	0	0	0	–	1
	Total	33	2	0	2	6	173	43
User type	Professional	67	0	0	5	6	–	78
	COVID-19 patient	64	5	4	1	2	–	76
	Relative	51	3	1	2	5	–	62
	Total	172	8	5	8	13	10	206

*USHC* University of Salamanca Healthcare Complex.

### Interventions, emotions, and symptoms

Among program participants, the most commonly reported symptoms were anxiety, insomnia, and crying (Table 3). At the end of the program, we did not monitor any subject, since we discharged or referred all users. Notably, during any of the interventions, we did not record substance abuse as a coping mechanism.

**Table 3:** Frequencies of interventions, emotions, and symptoms*.*

Interventios	Freq.	Emotions	Freq.	Symptoms	Freq.
Abreaction	170	Sadness	124	Anxiety	136
Self-care	142	Fear	99	Insomnia	114
Deactivation techniques	93	Impotence	66	Crying	93
Cognitive therapy	88	Exhaustion	59	Behavioral blocking/inhibition	61
Time management	88	Loneliness	51	Intrusive or repetitive thoughts	59
Awareness of personal resources	84	Lability	51	Isolation (rejects interaction)	43
Interaction encouragement	78	Anger	41	Somatization	42
Revaluation	74	Frustration	36	Lost appetite	42
Bereavement counselling	53	Irritability	32	Hyperactivity/restlessness	31
Scientific information	36	Blame	22	Interpersonal conflict	31
Prevention of suicidal ideation	6	Anxiety	11	Efforts not to think / not feel	22
				Mental confusion	11
				Ideas of death	10
				Clinophilia	9
				Obsessions	8
				Compulsive acts	7
				Distrust	7
				Altered appetite (excess)	6
				Dissociative symptoms or denial	5
				Self harm	2
				Suicidal ideas	2
				Substance abuse	0

We observed no significant differences in the level of care provided to participants based on their marital status (*p* = 0.076), employment situation (*p* = 0.414), relationship with a COVID-19 patient (*p* = 0.194), or profession (e.g., doctor, nurse, clinical assistant, or other healthcare professions) (*p* = 0.342).

## Discussion

The PASMICOR program successfully addressed the increased demand for mental health services resulting from the COVID-19 pandemic in the province of Salamanca. Since 79.6% of participants received comprehensive care through the program without requiring referrals to other services, we consider that the program effectively met the majority of participants’ needs.

It is notable that COVID-19 patients, family members, and healthcare workers participated in the program in similar proportions. Healthcare workers were the most numerous group, which may be due to targeted advertising in healthcare facilities and to this group being particularly affected by mental health issues related to the COVID-19 pandemic.

Consistent with previous research^20^, men were less likely to seek mental health care through the program. This suggests that we may need a more proactive approach to encourage men to seek help when needed^21^. One possible explanation for why female healthcare workers received more care through the program is that women are overrepresented in healthcare professions. All of this leads to gender disparities being sustained and increasing with crises^22^.

The age distribution of program participants did not align with the population group most affected by the COVID-19 pandemic^23^, due to the specific inclusion criteria of the program mentioning before. Few participants were residents of nursing homes, as their symptoms were not severe enough to require mental health treatment. We did not include healthcare workers over the age of 65 in the program, as they exceeded the retirement age limit. However, there was greater variability in the ages of family members, as we applied no age restrictions to this group.

We initiated the PASMICOR program in response to increased demand for mental health services in order to avoid an overbooking of new patiens in the already existing mental health network. The program began after a few weeks as a rapid response to the health emergency. Despite this, the severity of the symptoms found was high, showing that it was high from the beginning as observed in other mental health interventions^24–27^. As a free intervention, the program removed access barriers for vulnerable communities^25^. Other studies have highlighted social isolation and worsening pre-existing mental disorders as key issues during the pandemic^24^. Depression, anxiety, and stress are common emotions experienced during this time^28^, and while recovery from these symptoms has been observed, overall mental health remains poor among many individuals^29^. Globally, first reports indicates that suicide rates did not increase during the COVID-19 pandemic, but there was a higher lethality among 65 years old retired employment males^30–32^. We corroborate findings from other studies^33^ that the utilization of telemedicine proves successful, with no specific complaints from users or program professionals. This underscores the effectiveness of non-face-to-face interventions, which not only maintain effectiveness but also mitigate the risk of COVID-19 transmission. Additionally, in our case, telemedicine interventions could explain why we recorded no completed suicides or low rates of suicidal thoughts or ideation among program participants. Another possible explanation is that we referred individuals at risk for suicide to psychiatric care. As limitations of this study, the types of therapy or the approach offered may have varied depending on the group or on the psychologist assigned to each patient. Because healthcare professionals facilitated access to the program during an emergency situation, it is possible that only extreme cases were referred for treatment, as some individuals may have avoided seeking care at healthcare facilities due to fear of contagion. All referred patients were able to speak, so there were not severe COVID-19 cases. The absence of prior mental health recordkeeping could have influenced the study outcomes, potentially skewing the results. Finally, it is noteworthy that the program was only applied in Salamanca, being configured as a single area, and it is possible that the mental health network was different in other areas of the country. However, this is a real-world experience that should be taken into account for planning. It provides an immediate and medium-term response to critical situations such as a pandemic and shows that few patients were readmitted or referred to the mental health network.

This study provides valuable information on the demand for real word mental health care during a national emergency situation and on common symptoms and emotions experienced by individuals affected by COVID-19 without filters. It also provides insight into the types of interventions used and the personnel and time required to meet demand. We found telepsychology to be an effective form of care delivery that could prevent the normal mental health network from collapsing. We were able to discharge a small portion of the patients progressively. Since few participants required repeat treatment, we can consider that the program successfully met their needs. Similar programs have been implemented elsewhere, but data on their management and effectiveness are not available. We point out that we referred few participants to standard mental health services when the program ended.

As recommendations for improvement, we suggest assigning dedicated staff to the program from the start to avoid overloading professionals who are also responsible for other duties. It would be useful providing adequate training to psychologists could help shorten intervention duration, and increase the number of patients served. Additionally, providing therapy for program staff, such as defusing and debriefing, may also be beneficial. For future interventions, it may be beneficial to focus more on women and other groups most affected by the pandemic.

### Supplementary Information

Supplementary Information 1.

## Data Availability

Data are available from the correspondence author, upon request. Data are not publicly available due to ethical issues.
