# Supplementary material for: Follow-up of telemedicine mental health interventions amid COVID-19 pandemic
Source: Sci Rep. 2024 Jun 28;14:14921. doi: 10.1038/s41598-024-65382-w (PMC11213890; doi:10.1038/s41598-024-65382-w)
Supplement: Supplementary file 1 — Supplementary Information 1. [file 41598_2024_65382_MOESM1_ESM.docx]

PASMICOR

| Date |  | DD/MM/YYYY | | | | | |  |
| --- | --- | --- | --- | --- | --- | --- | --- | --- |
| ID | Identification | Medical History No. | | | | | |  |
|  |  | Failing that: acronyms of **name and surname** | | | | | |  |
| Demographic | Sex | Male (1), Female (2) | | | | | |  |
|  | Age | Age:  1: 0-**1**0 6: 51-**6**0  2: 11-**2**0 7: 61-**7**0  3: 21-**3**0 8: 71-**8**0  4: 31-**4**0 9: 81-**9**0  5: 41-**5**0 10: 91 onwards | | | | | |  |
|  | Marital status | Single. (1); In a relationship. (2); Widower. (3); Separated (4) | | | | | |  |
|  | Profession | Physician. (1); Nurse. (2); Clinical assistant. (3); Healthcare worker. (4); Other(5) | | | | | |  |
|  | Employment Status | Active. (1) On sick leave. (2) Retired (3) Others (indicate if known) | | | | | |  |
| Indicate the personal situations of the attended user (at the time of referred) If present, it is coded with (1), if it does not present, it is left blank. | | | | | | | |  |
| Personal situation | Does the individual provide or provided professional assistance to COVID-19 patients? | | | | | | |  |
|  | Patient [If present, it is coded with (1), if it does not present, it is left blank]. | | | Not hospitalized | | | |  |
|  |  |  |  | Currently hospitalized for COVID-19 for **1-7 days** | | | |  |
|  |  |  |  | Currently hospitalized for COVID-19 for **more than 7 days** | | | |  |
|  |  |  |  | Isolated at home | | | |  |
|  |  |  |  | Admitted to a **hotel** or similar | | | |  |
|  |  |  |  | Admitted to a **geriatric** residence | | | |  |
| Familiar | Family [If present, it is coded with (1), if it does not present, it is left blank]. | | Has family members hospitalized due to COVID-19 for **1-3** days. | | | | |  |
|  |  |  | Has family members hospitalized due to COVID-19 for **4-7** days. | | | | |  |
|  |  |  | Has family members hospitalized due to COVID-19 for **more than 7** days. | | | | |  |
|  |  |  | Has close relatives who have **died** in the previous days | | | | |  |
|  | Kinship [If present, it is coded with (1), if it does not present, it is left blank]. | | | | | Partner | |  |
|  |  |  |  |  |  | Father | |  |
|  |  |  |  |  |  | Mother | |  |
|  |  |  |  |  |  | Brother | |  |
|  |  |  |  |  |  | Son | |  |
|  |  |  |  |  |  | Other (specify) | |  |
| Level | Level I or II | | | | | | Complexity level |  |
|  | How is the subject classified? | | | | CAUSA (1), Primary Care (2), Nursing Homes (3). | | |  |
|  | How was the original contact made? | | | | Email (1), informal (2). | | |  |

| Predominant Symptoms | [If presented, it is coded with (1), if it does not present, it is left blank.] | | | | Behavioral Blocking/blockage | |  |
| --- | --- | --- | --- | --- | --- | --- | --- |
|  |  |  |  |  | Hyperactivity/restlessness | |  |
|  |  |  |  |  | Clinophilia | |  |
|  |  |  |  |  | Isolation (rejects interaction) | |  |
|  |  |  |  |  | Interpersonal conflict | |  |
|  |  |  |  |  | Substance abuse | |  |
|  |  |  |  |  | Compulsive acts | |  |
|  |  |  |  |  | Self-harm | |  |
|  |  |  |  |  | Crying | |  |
|  |  |  |  |  | Somatic symptoms/somatization | |  |
|  |  |  |  |  | Insomnia | |  |
|  |  |  |  |  | Loss of appetite | |  |
|  |  |  |  |  | Altered appetite (excess) | |  |
|  |  |  |  |  | Anxiety | |  |
|  |  |  |  |  | Intrusive repetitive thoughts | |  |
|  |  |  |  |  | Dissociative Symptoms/Denial... | |  |
|  |  |  |  |  | Efforts not to think/feeling | |  |
|  |  |  |  |  | Mental confusion | |  |
|  |  |  |  |  | Distrust | |  |
|  |  |  |  |  | Ideas of Death | |  |
|  |  |  |  |  | Suicidal ideation | |  |
|  |  |  |  |  | Obsessions | |  |
|  |  |  |  |  | Other (specify) | |  |
| Predominant Emotional Experience | (Indicate the top 3 most relevant in the patient 1st, 2nd, 3rd) | | | | | Fear |  |
|  |  |  |  |  |  | Guilt |  |
|  |  |  |  |  |  | Anger |  |
|  |  |  |  |  |  | Irritability |  |
|  |  |  |  |  |  | Sadness |  |
|  |  |  |  |  |  | Loneliness |  |
|  |  |  |  |  |  | Lability |  |
|  |  |  |  |  |  | Impotence |  |
|  |  |  |  |  |  | Frustration |  |
|  |  |  |  |  |  | Exhaustion |  |
|  |  |  |  |  |  | Others (specify which and their position) |  |
| Intervention | [If presented, it is coded with (1), if it does not present, it is left blank.] | | | Emotional Facilitation/Abreaction | | |  |
|  |  |  |  | Cognitive Therapy | | |  |
|  |  |  |  | Explaining self-resources | | |  |
|  |  |  |  | Revaluation | | |  |
|  |  |  |  | Self-care | | |  |
|  |  |  |  | Encourage Interaction | | |  |
|  |  |  |  | Deactivation, self-regulation, *mindfulness techniques* | | |  |
|  |  |  |  | Time Management – Behavioral Activation | | |  |
|  |  |  |  | Support in grieving | | |  |
|  |  |  |  | Scientific-health information | | |  |
|  |  |  |  | Guidelines for self-harm containment | | |  |
| Evolution | [If presented, it is coded with (1), if it does not present, it is left blank.] | | Discharge (favorable progress) | | | |  |
|  |  |  | Rejects care | | | |  |
|  |  |  | Referral to psychiatric care | | | |  |
|  |  |  | Referral to a psychologist from our mental health team | | | |  |
|  |  |  | In PASMICOR follow-up | | | |  |
|  |  |  | Exitus | | | |  |
| Calls |  | Number. | | | | |  |
|  |  | Total estimated duration of calls, in minutes. | | | | |  |
| Notes |  | | | | | |  |
